# An algorithm for the early diagnosis and correct approach to dementia management: results of a multiprofessional team

**DOI:** 10.1007/s40520-024-02749-z

**Published:** 2024-05-03

**Authors:** Pietro Gareri, Antonino Maria Cotroneo, Giovanni Gelmini, Enrico Mossello, Massimiliano Massaia, The Touch Working Group, Francesca Bartalucci, Lorenzo Bellintani, Erika Cerracchio, Maurizio Corsi, Sara Duca, Natasa Dikova, Carlo Fattorelli Salimbeni, Antonina Gorizia, Chahariar Makoui, Marco Mantovani, Vanda Menon, Maria Modugno, Alessandra Nesti, Paolo Paolello, Chiara Perrone, Riccardo Risaliti, Rosa Aura Scarpinato, Fabrizio Scataglini, Enrico Vitale, Loredana Zanini, Rosa Abbruzzese, Luciano Castagna, Raffaele Conforti, Stefano Crooke, Giovanni Dragoni, Giuliana Fabbri, Fraia Falvo, Roberta Frezza, Maria Paola Gozzoli, Jasmine Invernizzi, Marta Lagorio, Antonio Lalli, Cristina Miceli, Rossella Obialero, Valentino Olivelli, Paolo Raganato, Ernesto Rampin, Graziella Rossi, Stefania Speme, Cristina Triches, Maria Villanova, Martina Balducci, Viera Boccuti, Roberta Chiloiro, Lucilla Colarusso, Francesca Crosta, Licia D’Andrea, Eleonora Greco, Maria Pia Iovenitti, Nunziata Leopardi, Chiara Marchini, Carmina Menza, Rosalba Patella, Monica Pugnotti, Riccardo Rapelli, Giulia Sinibaldi, Giovanna Alagona, Sebastiano Arena, Vito Maria Bagnulo, Valentina Baiamonte, Marco Burrascano, Salvatore Chessari, Eleonora Rita Chiarenza, Margherita Chirico, Floriana Crivello, Anna Di Prima, Angelo Di Stefano, Salvatore Dieli, Rosalba Ferrigno, Angelo Garifoli, Luigi Marrucci, Domenico Martelli, Antonio Nieddu, Epifanio Rapisarda, Maria Rosaria Sateriale, Claudia Scalise, Amedeo Venezia, Vincenzo Zupo, Luciana Attino, Barbara Barbato, Linda Berton, Roberto Chiesa, Antonio Colin, Emma Comitale, Lino De Angelis, Luigi De Mitri, Carlo Fagherazzi, Teresa Falco, Stefania Ferracin, Giuseppe Leone, Antonio Maddonni, Paola Mainquà, Maria Melfi, Carolina Anna Mobilia, Antonio Montella, Linda Morselli, Marco Mosele, Giulia Pelagalli, Maria Pratesi, Gianfranco Puzio, Gian Luca Simonini

**Affiliations:** 1 Department of Frailty, Head Physician CDCD Catanzaro Lido – ASP Catanzaro, Viale Crotone 214, 88100 Catanzaro, Italy; 2 Director Complex Geriatric Unit Maria Vittoria Hospital Turin, Turin, Italy; 3 Director South East District, AUSL, Parma, Italy; 4 Department of Experimental and Clinical Medicine, University of Florence and SOD Geriatrics-UTIG, AOU Careggi, Florence, Italy; 5 Head Physician CDCD, Complex Unit of University Geriatrics - AO Health and Science City, Turin, Italy

**Keywords:** Early diagnosis, Dementia, Multi-professional team, Older people, Treatment

## Abstract

**Backgroung:**

The early identification of cognitive disorder is a primary scope, because it could reduce the rate of severe cognitive impairment and thus contribute to reduce healthcare costs in the next future.

**Aims:**

The present paper aimed to build a virtuous diagnostic path of cognitive impairment, highlighting all the professionalism that can serve this purpose.

**Methods:**

The Delphi method was used by the experts, who reviewed the information available during each meeting related to the following topics: early diagnosis of cognitive impairment, definition of Mild Cognitive Impairment, unmet needs in post-stroke patients, critical decision-making nodes in complex patients, risk factors, neuropsychological, imaging diagnosis, blood tests, the criteria for differential diagnosis and the possible treatments.

**Results:**

The discussion panels analyzed and discussed the available evidences on these topics and the related items. At each meeting, the activities aimed at the creation of a diagnostic-welfare flow chart derived from the proposal of the board and the suggestions of the respondents. Subsequently, the conclusions of each panel were written, and the study group reviewed them until a global consensus was reached. Once this process was completed, the preparation of the final document was carried out.

**Conclusions:**

Eventually, we built an algorithm for the early diagnosis and treatment, the risk factors, with the possible differences among the different kinds of dementia.

## Introduction

The prevalence of Alzheimer’s disease (AD) has been increasing in recent years and we expect to go from the about 5.8 million Americans aged 65 years and older in 2020 (80% out of them aged 75 years old or older) [1] up to 13.8 million individuals in 2050 [2]. In 2019, the incidence of Alzheimer's disease and other dementias in Europe was approximately 188 per 100,000. Over the provided time interval, the incidence of Alzheimer's disease has increased in Europe. The prevalence of Alzheimer's disease in Europe was estimated at 5.05%, and increased with age. The incidence of Alzheimer's disease in Europe was 11.08 per 1000 person-years (95% CI, 10.30–11.89). These rates increased with age. It is estimated that there will be around 82 million people suffering from dementia in 2030 [3].

Henceforth, the importance of early identification of cognitive disorder becomes a primary scope, to start non-pharmacological measures and specific therapeutic treatments aimed at slowing down the evolution of dementia. Indeed, early detection could reduce the rate of severe cognitive impairment and thus contribute to reduced healthcare costs in the next future.

The present paper aimed to build a virtuous diagnostic path of cognitive impairment, highlighting all the professionalism that can serve this purpose.

We will therefore build an algorithm for early diagnosis and treatment, with the possible differences among the different kinds of dementia.

Early diagnosis of cognitive impairment requires the administration of neuropsychological tests, blood tests and brain imaging. There are some tips which can be used for a correct diagnosis, and we will give some suggestions for it. For example, diagnosis of AD could be made according to clinical symptoms, neuropsychological tests (Mini Mental State Examination, Montreal Canadian Assessment, NINCDS-ADRDA), the possible presence of hypometabolism of the temporoparietal cortex and posterior cingulate/precuneus cortex on 18-fluorodeoxyglucose – PET (FDG PET) and/or the presence of atrophy of the medial temporal lobe on brain imaging (CT scan or MRI scan) [4]. The role of amyloid PET imaging in AD diagnosis is becoming more and more remarkable [5].

Diagnosis of mixed dementia is made whenever symptoms that are usually typical of AD such as memory loss are associated with symptoms due to cerebrovascular deficits, i.e., impaired judgement, ability to make decisions, plan or organize [6]. When vascular cognitive impairment is more evident, brain imaging usually shows atrophy together with brain injuries such as multiple microinfarcts/old infarcts, lacunar infarcts in strategically placed infarcts, periventricular white matter hyperintensities or hypodensities, microscopic bleeding [6]. However, we will have the opportunity of deepening these aspects in the article.

## Methods

The study was planned by a scientific board made up of medical doctors specialized in the care and management of people suffering from dementia. The participants were selected according to their expertise in the field of dementia. All of them were involved in their clinical practice in Centers for Cognitive impairment and Dementia all over Italy. All of them were used to treat patients affected by dementia on a daily basis.

Five meetings were held in different parts of Italy (Padua, Ancona, Rome, Florence, Bologna), with an audience of geriatricians and neurologists.

The Delphi method was used by the experts, who reviewed the information available during each meeting related to the following topics: early diagnosis of cognitive impairment, definition of Mild Cognitive Impairment, unmet needs in post-stroke patients, critical decision-making nodes in complex patients, risk factors, neuropsychological, imaging diagnosis, blood tests, the criteria for differential diagnosis and the possible treatments [7]. Delphi methods can be defined as a structured technique to modulate a group communication process effectively in allowing a group of individuals to deal with a complex problem. It is now a widely used method to generate group consensus, and develop qualitative practice points. A four-step methodological process with nine qualitative evaluation points was defined in a recent article (1. Problem area – systematic identification of problem area; 2. Panel members – selection based on objective and a predefined criteria; 3. Delphi rounds – anonymity of panelists and responses; controlled feedbacks and iterative rounds; 4. Closing criteria – consensus criteria; analysis of consensus; closing criteria defined a priori; stability of results) [8].

The discussion panels analyzed and discussed the available evidences on these topics and the related items. At each meeting, the activities aimed at the creation of a diagnostic-welfare flow chart derived from the proposal of the board and the suggestions of the respondents. Subsequently, the conclusions of each panel were written, and the study group reviewed them until a global consensus was reached. Once this process was completed, the preparation of the final document was carried out.

Eventually, we built an algorithm for the early diagnosis and treatment, the risk factors, with the possible differences among the different kinds of dementia.

The discussion started from three presentations regarding mild cognitive impairment (MCI), the unmet needs in post-stroke patients and the critical decision-making nodes in complex patients; two clinical case reports were also presented and served for promoting an active discussion with the audience, starting from the possible early detection through the general practitioner and going through the role of Centers for Cognitive Impairment and dementia for 1st and 2nd level tests.

The board then met again to come to a conclusion that would take into account the considerations/suggestions of colleagues who had been present at the meetings and definitely for building a proposal of algorithm which could be used for making a diagnosis of dementia of any kind easier.

The working group discussed about blood tests, for example blood count, folate, vitamin D, vitamin B12, thyroid function, and homocysteine. Among the neuropsychological tests, the first test to be administered by the general practitioner (GP) was meant the GPCOG (General Practitioner Cognitive Test). Furthermore, in the Centers for Cognitive Impairment and Dementia (CDCD), first and second level tests are usually performed (Table 1).

**Table 1:** Second level tests for detecting cognitive impairment

Memory
Rey’s word test
Verbal span
Corsi test
Babcock test
Rey complex Figure
Language
Phonetic and semantic fluency
Praxis
Copy freehand drawings
Clock drawing test
Copy of rey’s complex figure
Attention
Attentional matrices
Stroop’s test
Executive funcions
Wisconsin’s test
Trail making test–A (TMT-A)
Trail making test–B (TMT-B)
Frontal Assessment Battery (FAB)
Intelligence
Raven’s progressive matrices

The GPCOG is a short test made up of a cognitive test of the patient (requiring about four minutes) and a two minutes informant interview. Both the cognitive test and the interview can be scored separately, together, or sequentially [9]. The cognitive test includes nine items and the maximum score is 9 (fewer points indicate more impairment) [9]. The informant interview increases the predictive power because it regards six historical questions from an informant/next of kin to whom is asked to compare the patient's current performance (including memory, word finding difficulties, difficulties in managing finances and/or medication independently and needing assistance with transportation) with his/her function a few years ago [9].

The first level tests are the MMSE (Mini Mental State Examination Test) and MoCA (Montreal Canadian) tests; the MMSE test is the gold standard for large scale investigations of cognition and memory functions overall. The MoCA test is a brief test of cognitive functions, taking 10 min when administered by an expert person [10, 11]. It has been considered as an alternative to the MMSE [12] as a screening test for non‐specialist use since the latter is now copyrighted and there is a charge for its use. Furthermore, it offers more detailed testing of executive function [13]. Indeed, it assesses executive function, attention, concentration and working memory, short‐term memory, language, and orientation.

Table 1 reports the second level tests commonly used for deeper investigations on memory functions [14].

## Results

It was proceeded to examine step by step the various contents related to the diagnosis of MCI, mild, moderate and severe dementia, with the indication of diagnostic and care paths.

**Fig. 1:**
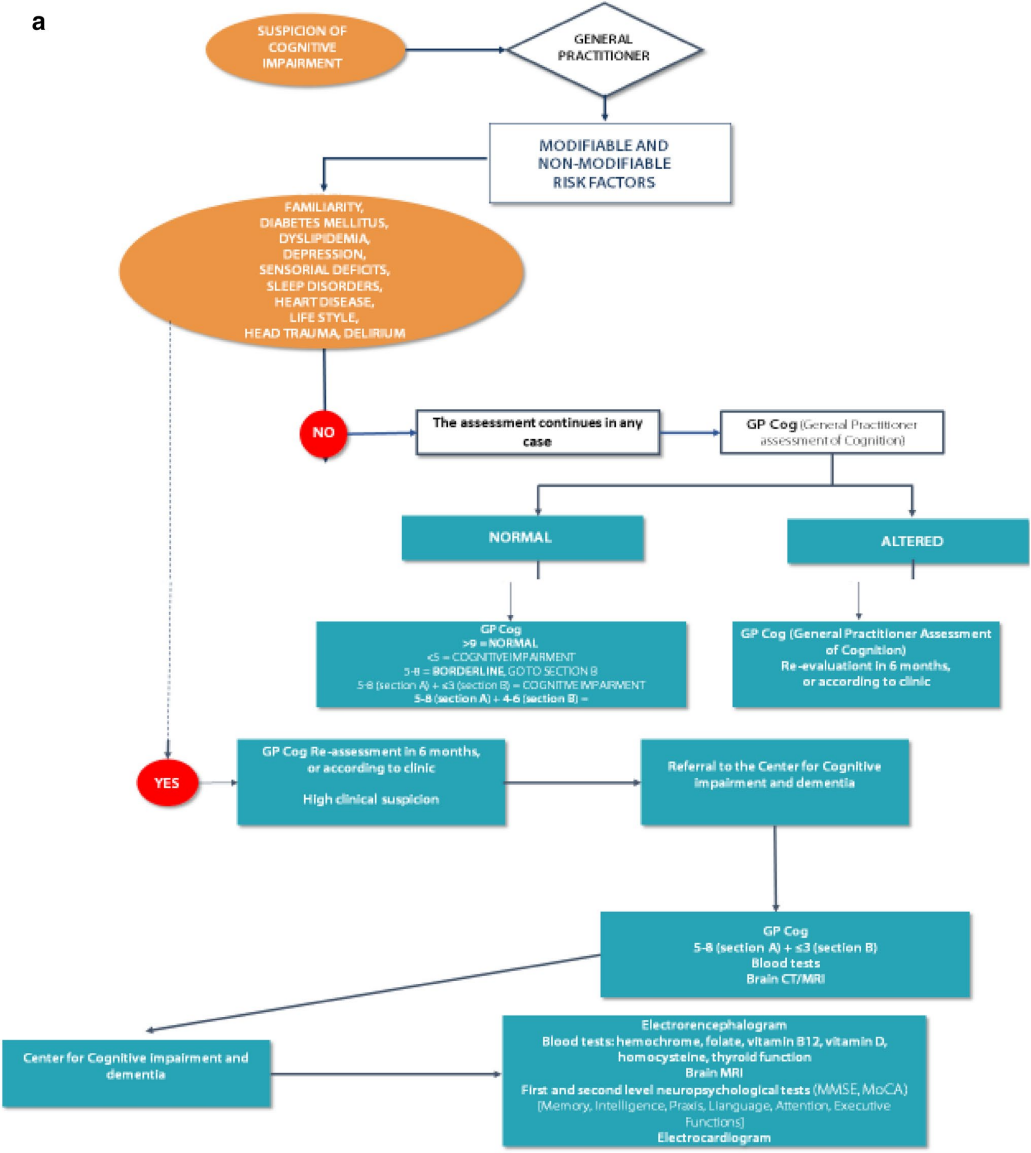

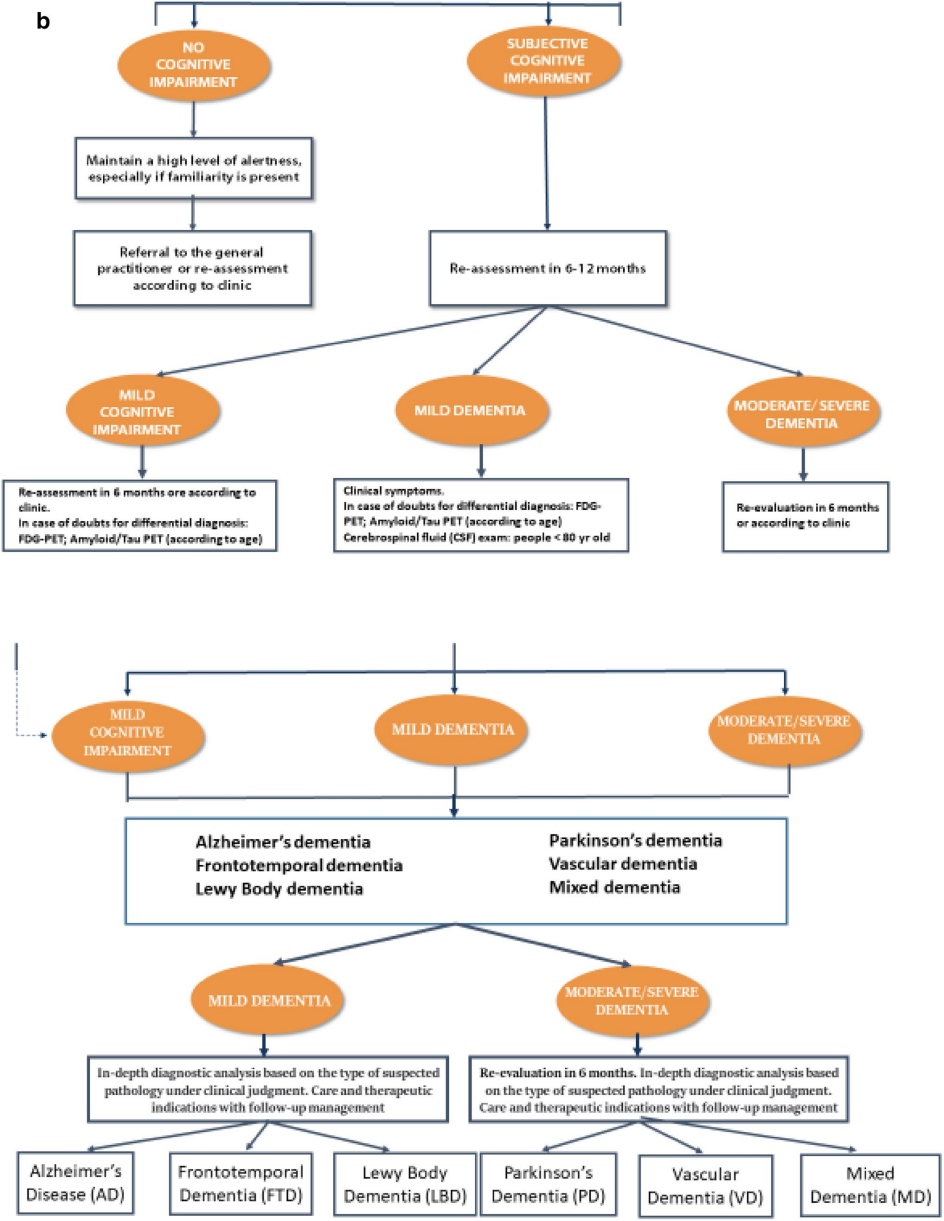

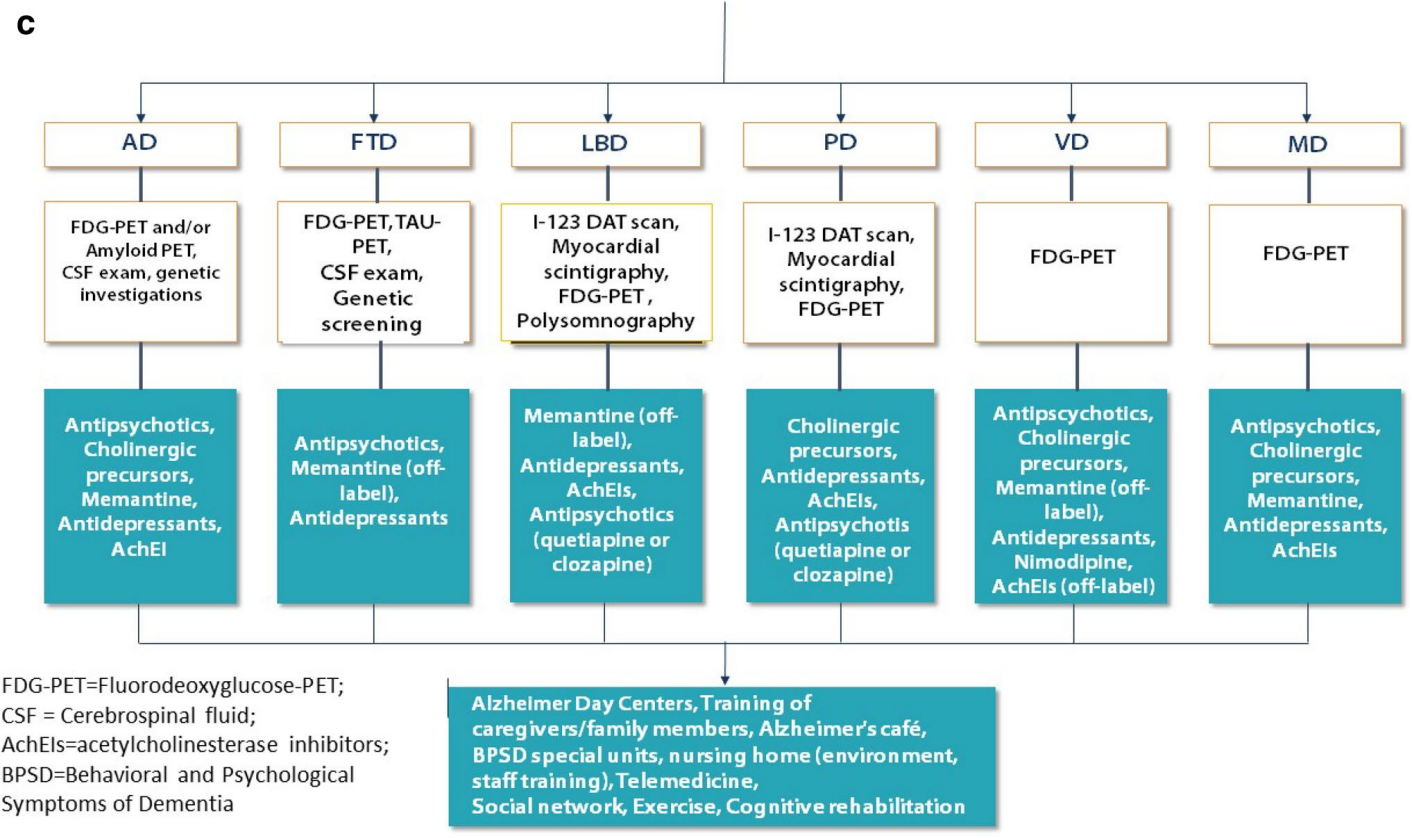
**a** The first steps: the roles of general practitioners and Centers for Cognitive Impairment and Dementia. **b** The figure shows the management of people with cognitive impairment, from Mild Cognitive Impairment up to Dementia. **c** Kinds of dementia and the possible treatments

We report in details the desirable paths (Fig. 1a, b, c).

The first step when facing a clinical suspicion or cognitive impairment is the administration by the general practitioner of the GPCoG test (General Practitioner Cognitive Test). The GP intuition is a remarkable point to focus on for a whatever diagnosis of cognitive impairment/dementia .

In the steps ahead, we have tried to build an algorithm; with a cascade mechanism, starting from the suspicion of the general practitioner and continuing with all the exams that can be requested after a specialistic approach, we arrived at a differential diagnosis among the various kinds of dementia. We explained how to arrive at the diagnosis and tempted a therapeutic approach, without neglecting the possible care places where better the multifaceted aspects of dementia can be faced.

## Discussion and conclusions

Creating an algorithm can help health professionals involved in the field of dementia, unraveling excessively complex skeins, especially if patients are old, frail, and poly-treated.

Waiting for a truly decisive drug in the treatment of dementia, it is also necessary to use all the therapeutic paraphernalia available today.

It is important that general practitioners are made aware of early detection of cognitive impairment; the GPCog was accepted as an easy tool to this purpose. It takes between two to five minutes to administer the GPCOG and it does not require the administrator to have extensive training. It has been translated into several languages, but there is not enough research on how different cultures and languages might affect the test's results. Furthermore, it requires the physical ability to write or draw. Therefore, if a person is not able to hold a pen or pencil, he/she is unable to complete the clock drawing test.

The suspicion of cognitive impairment is remarkable for going beyond a fast assessment, thus avoiding an ageistic judgment of a “normal age-related impairment”. Also, as suggested by the common clinical practice and by the working group, another remarkable step is the assessment of the most frequent risk factors. The working group agreed to maintain a high level of alertness, especially if familiarity is present. Then, there was an intense discussion on diagnostic tools, especially neuropsychological tests, and instrumental exams, especially for giving strength to the possible solutions for differential diagnosis.

Among the instrumental tools, CT and MRI scans are routinely used. MRI scan gives more information on some crucial structures of the brain, for example, the hippocampus volume. FDG-PET is regarded as an effective biomarker for earlier diagnosis of AD [15]. Patients with AD tend to show hypometabolism on 18-FDG-PET scan in the regions of the posterior cingulate, parietotemporal cortices, and frontal lobes [15]. It is worthwhile and cost effective to use it when there are some uncertainties in diagnosis.

Amyloid PET is a reliable diagnostic imaging tool, and its use should be encouraged for early differential diagnosis in clinical settings and for selecting patients for disease-specific therapies [16]. In the last years, its use has become more and more increasing; this is good from one side, because diagnoses are more accurate. On the contrary, Tau PET is not for routine use; it has been shown to be a promising tool for predicting cognitive change that is superior to amyloid PET and MRI and may support the prognostic process in preclinical and prodromal stages of AD [17].

Other instrumental tools can be used according to the clinical symptoms: for example, I-123 DAT scan for PD diagnosis, myocardial scintigraphy for differential diagnosis of PD, and atypical Parkinsonism. Polysomnography is cost effective in LBD, where it can be used for characterizing different findings in patients reporting sleep-related complaints [18].

Furthermore, genetic screening must be used only if there are clear suspicions of hereditary.

In the treatment of cognitive disorders, cholinergic precursors, as demonstrated by the ASCOMALVA study with choline alphoscerate, in association with donepezil [19] and studies such as the CITIRIVAD, CITICHOLINAGE, CITIMEM, CITIDEMAGE, CITIMERIVA, CITIMEA, carried on with citicoline in association with AchEIs and/or memantine, could serve to slow the evolution of the disease [6, 20–24]. The treatment of behavioral and mood disorders requires the right dosage/benefit ratio between non-pharmacological and pharmacological therapies [25, 26]. Therefore, antidepressants and antipsychotics are still important for treating some typical characters of dementia, such as depression, agitation, sleep disorders, anxiety, sundowning, and so on [27, 28].

Far from ancillary, the support of the structures used for the care of the person (day centers, dementia nuclei, etc.) is remarkable, especially in the forms of moderate to severe dementias.

The novelty derives from a shared discussion in all the steps mentioned above, from diagnosis to therapeutic decisions. It is what occurred in the five meetings where medical doctors, all involved in the field of dementia, discussed and found solutions to all the queries that could be raised. We think that this can offer the reader an easy tool for navigating through the complex field of dementias.

Collecting all the ideas and experiences of a group of a multi-professional team for each meeting and then rearrange all of them in a final document required a big effort. We tried to share a document that can concretely resume all the steps to move from diagnosis to treatment and places of care for people affected by dementia.

The limits of the present paper are linked to the fact that the algorithm was built upon the opinion of experts; it was not used a GRADE system (grading of recommendations, assessment, development and evaluation). The GRADE system started in 2000 as an informal collaboration among people, with the objective of developing a common and rational method for grading the quality of evidence and the strength of recommendations. It lets a comprehensive approach for improving or disqualifying the quality of evidence, a transparent process for translating the evidence into recommendations, and a clear and pragmatic interpretation of the strengths and weaknesses of the recommendations for clinicians, patients and all the people responsible of health policies.

However, a positive point to focus on is that the present algorithm was built through the opinion of more than one hundred experts in the field of dementia and the wise and careful assessment of the coordinator group, which significantly contributed to the drafting of the final paper.
